# Can Cryptogonimids of the Same Genus Influence Each Other’s Level of Genetic Variation?

**DOI:** 10.3390/biology14010006

**Published:** 2024-12-24

**Authors:** Ekaterina S. Tokarskaya, Yulia V. Tatonova, Haneef Ahmed Amissah, Polina G. Shumenko, Mikhail Yu. Shchelkanov

**Affiliations:** 1 Federal Scientific Center of the East Asia Terrestrial Biodiversity, Far Eastern Branch, Russian Academy of Sciences, Vladivostok 690022, Russia; 2 Institute of Life Sciences and Biomedicine, Department of Medical Biology and Biotechnology, School of Medicine and Life Sciences, Far Eastern Federal University, Vladivostok 690922, Russia; 3 Diagnostics Laboratory Department, Trauma and Specialist Hospital, CE-122-2486, Central Region, Winneba P.O. Box 326, Ghana; 4 G.P. Somov Research Institute of Epidemiology and Microbiology, Russian Federal Service for Surveillance on Consumer Rights Protection and Human Wellbeing, Vladivostok 690087, Russia; 5 Institute of Life Sciences and Biomedicine, Basic Department of Epidemiology, Microbiology and Parasitology School of Medicine and Life Sciences, Far Eastern Federal University, Vladivostok 690922, Russia

**Keywords:** *Exorchis oviformis*, *Exorchis convictus*, Cryptogonimidae, genetic diversity, competitive relationship

## Abstract

Trematodes of the genus *Exorchis* are parasites that are common in East and Southeast Asia. These parasites are known to infect fish, thus impacting aquaculture significantly. An introspection into the dynamics of infection of these parasites has revealed possible co-infection in shared hosts. But data on the dynamics of their shared host on their population structure and genetic variability is lacking. In this study, we investigate the intraspecific structure of the *Exorchis* species, *Exorchis oviformis*, and *E. convictus*, both of which coexist in the same definitive host. By using partial gene sequences of the mitochondrial *cox1* gene (715 bp), including 30 newly obtained sequences from the southern part of the Russian Far East, we found a low level of genetic variation in both species, consistent with previous findings of other trematodes from the same region. We hypothesize that the observed low variability could be attributable to the process of host infection and transmission and abiotic factors influencing a recent decline in host variability and abundance. This is driving a strong selection pressure due to the limited number of parasites reaching the definitive host. This, among other factors, could possibly impact the long-term survival and adaptability of *Exorchis* and other trematodes at large in the region. However, we also believe that there may be a chance that these closely related species could exert an influence on each other’s genetic diversity, potentially limiting the variation within each species. To uncover the dynamics driving these observations, we propose the approach of expanding sampling, broader genetic marker analyses, and investigations of varied host populations that could elucidate the complexities of the *Exorchis* species genetic variability and evolutionary dynamics.

## Simple Summary

In this study, we investigate genetic variability in two closely related parasitic flatworms belonging to the genus *Exorchis* (Cryptogonimidae), *Exorchis oviformis*, and *E. convictus*, which infect commercially important catfish. By sequencing and analyzing the partial sequences of the *cox1* mtDNA gene of these species from the Russian Far East, we observe a surprising low genetic variability in both species. These unexpected findings could reflect the potential of these species to influence each other’s genetic diversity and, consequently, the parasite and host population dynamics.

## 1. Introduction

Trematodes of the genus *Exorchis* Kobayashi, 1921 (Cryptogonimidae) are widespread in East and Southeast Asia, with eight described species: *Exorchis oviformis* Kobayashi, 1918; *E. equistoma* Feng & Wang, 1997; *E. petalovaris* Feng & Wang, 1997; *E. macrobursae* Pan, 1984; *E. ovariolobularis* Cao, 1990; *E. multivitellaris* Pan, 1984; *E. dongtinghuensis* Zhang, Zuo, Liu & Zhou, 1993; and *E. convictus* Solodovnik, Tatonova, Urabe, Besprozvannykh, Nakao & Inoue, 2021 [1]. The first intermediate hosts of these parasites are mollusks of the families Stenothyridae Tryon, 1866; Bithyniidae Gray, 1857; and Pomatiopsidae W. Stimpson, 1865. Fish of the family Cyprinidae Rafinesque, 1815, serve as the second intermediate hosts, while the definitive hosts are fish of the family Siluridae Cuvier, 1816 [1,2,3]. The latter are the object of breeding in aquacultures for stocking purposes for recreational fisheries, as well as an economically important product in the catch and production system [4]. Metacercariae in the second intermediate host are localized in the muscles and gills, and less often in the fins. In the definitive hosts, these parasites reside in the small intestine [2]. Species of the genus *Exorchis* can have a significant impact on fish populations and aquaculture, causing reduced growth, increased morbidity, and economic losses in the aquaculture industry [5]. The results of a recent study in China showed a high level of infestation of *Silurus asotus* with parasites of the genus *Exorchis* [6]. According to the data, more than half of the Amur catfish (69.79%) were infested by representatives of the genus *Exorchis,* and the average intensity of infestation was 14.21 per fish. Therefore, a comprehensive study of this group of trematodes has, among other things, practical significance.

In a recent work devoted to the study of the genus *Exorchis*, the economic and ecological importance of understanding the population structure of these parasites has been underscored, particularly given that *Exorchis oviformis* and *Exorchis convictus* can co-parasitize the same definitive host [1]. Studying the population structure of both parasites is crucial to comprehending the microevolutionary processes that have occurred in the past. In the same vein, it gives us the opportunity to understand the current state of the species and will enable the continued monitoring of the spread of these trematodes. However, data on the genetic diversity and population structure of these trematodes remains insufficient, with unanswered questions regarding the potential influence of shared hosts on their genetic variation. Thus, this study aims to investigate the genetic diversity and population structure of *Exorchis oviformis* and *Exorchis convictus*, using partial *cox1* mtDNA gene sequences and highlighting the potential impact of their shared hosts on their genetic variation. A previous observation is a recent decrease in host abundance driving a strong selection pressure due to the limited number of parasites reaching the definitive host. The data suggest the need for extended studies with a larger sample size and geographical span to be conducted. This current study contributes to our understanding of the evolutionary dynamics of *Exorchis oviformis* and *Exorchis convictus*, ultimately providing information on their population structure and their impact on natural populations.

## 2. Materials and Methods

Adult worms of *Exorchis oviformis* and *E. convictus* were harvested from the small intestines of three naturally infected catfish, *Silurus* sp. Linnaeus, 1758 (Actinopterygii: Siluridae), caught in two localities: the Razdolnaya River and the Elduga River (the right tributary of the Razdolnaya River). Two catfish from the Elduga River were dissected in 2022, and the third catfish from the Razdolnaya River was dissected in 2017. The obtained trematodes were washed in physiological solution, killed in distilled water, and fixed in 70% ethanol, then transferred to 96% ethanol.

Genomic DNA was isolated using the HotSHOT method [7]. Partial sequences of the *cox1* mtDNA gene were amplified using primers Co1-Fw (5′-GGGCAT-CCT-GAG-GTT-TAT-G-3′) and Co1-Rv (5′-AAC-AAATCA-TGA-TGC-AAA-AGG-TA-3′) [8]. The reaction mixture with a total volume of 10 µL contained 0.25 µL of each of the primers with a concentration of 10 mM, 5 µL of solution of GoTaq^®^ Green Master Mix (2×) (Promega Corporation, Madison, WI, USA), and 3 µL DNA. The sequencing reaction was performed using the same primers. The reaction mixture of 10 µL contained 1 µL of PCR product, 0.5 µL of primer, and 1 µL of Big Dye Terminator v3.1 (Applied Biosystems, Waltham, MA, USA). The nucleotide sequence was determined using the ABI 3500 Genetic Analyser (Applied Biosystems, USA; the Instrumental Centre for Biotechnology and Gene Engineering, FSCEATB FEB RAS).

The sequences were visualized in FinchTV 1.4.0, then assembled and aligned manually using MEGA 5.0 [9]. In addition to our own data, the sequences available in the NCBI database were also used in the analysis (Table 1). The genetic distances within and between species were determined with the MEGA program. Population analysis for the genus *Exorchis* species was performed using the Arlequin 3.11 [10] and DnaSP 5.10 [11] programs.

## 3. Results

**Figure 1:**
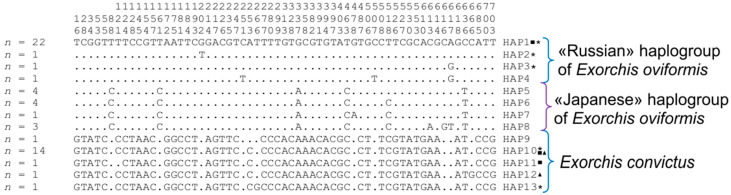
Variable sites of nucleotide sequences of the *cox1* gene for *Exorchis* spp. *Hap*—haplotype; *n*—number of sequences in the haplotypes. The numbers above the nucleotide sequences show the numbers of the variable sites (should be read vertically). Squares, triangles, and stars represent parasites from catfish 1 from the Elduga River, catfish 2 from the Elduga River, and catfish from the Razdolnaya River, respectively.

Thirty newly determined partial nucleotide sequences (715 bp) of the *cox1* gene were obtained and analyzed for two *Exorchis* species (12 and 18 nucleotide sequences for *E. convictus* and *E. oviformis*, respectively) from two localities in the Russian Far East. The total sample was 55 nucleotide sequences, including sequences available in NCBI, representing six geographic localities from Russia and Japan (Table 1). Eleven samples of *E. oviformis* and eight samples of *E. convictus* corresponded to the first catfish from the Elduga River. Five samples of *E. oviformis* and three samples of *E. convictus* were found in the second catfish from the Elduga River. Two samples of *E. oviformis* and one sample of *E. convictus* were removed from catfish caught in the Razdolnaya River. The sequences differ at 61 variable sites, of which 54 are parsimony-informative (Figure 1).

**Figure 2:**
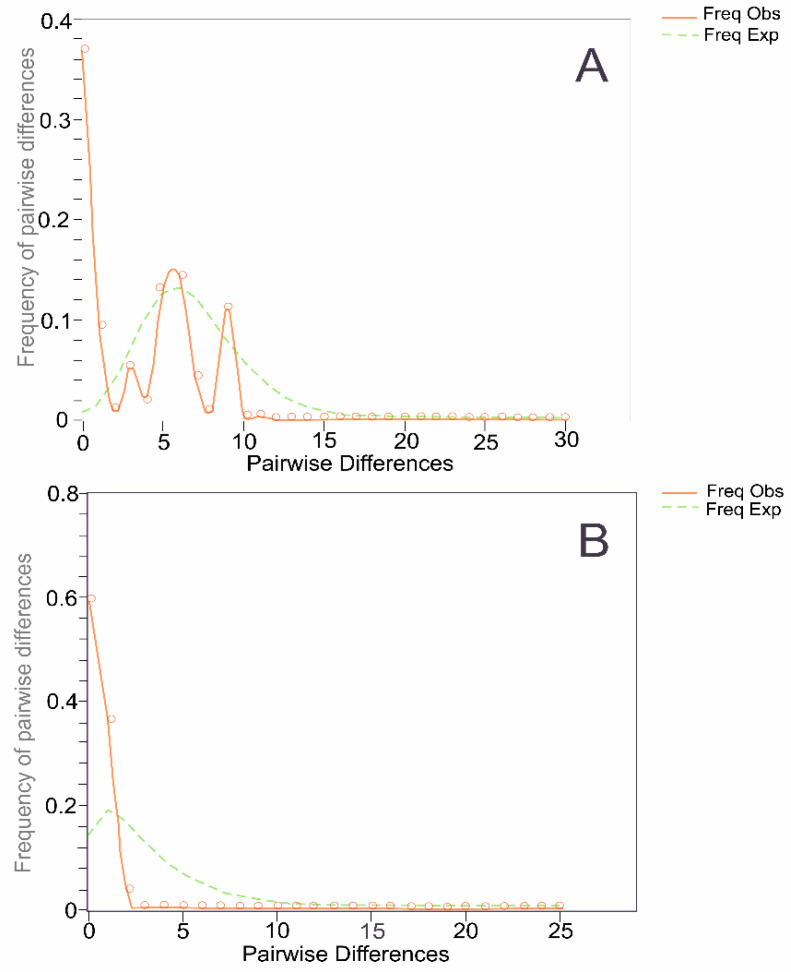
Mismatch distribution graphs for the partial sequence of the *cox1* mtDNA gene of *Exorchis oviformis* (**A**) and *Exorchis convictus* (**B**). *Exp*, expected distribution, *Obs*, observed distribution.

Genetic distances (*p*-distances) between and within species are presented in percentages for easier visualization and comparison with cited studies [1]. The genetic distances of 0.1 and 0.5% were observed within the *E. convictus* and *E. oviformis*, respectively, while the difference between the species was 7.1%. The mismatch distribution graph showed a multimodal shape for *E. oviformis*, while the graph for *E. convictus* has a unimodal L-shaped distribution (Figure 2).

**Figure 3:**
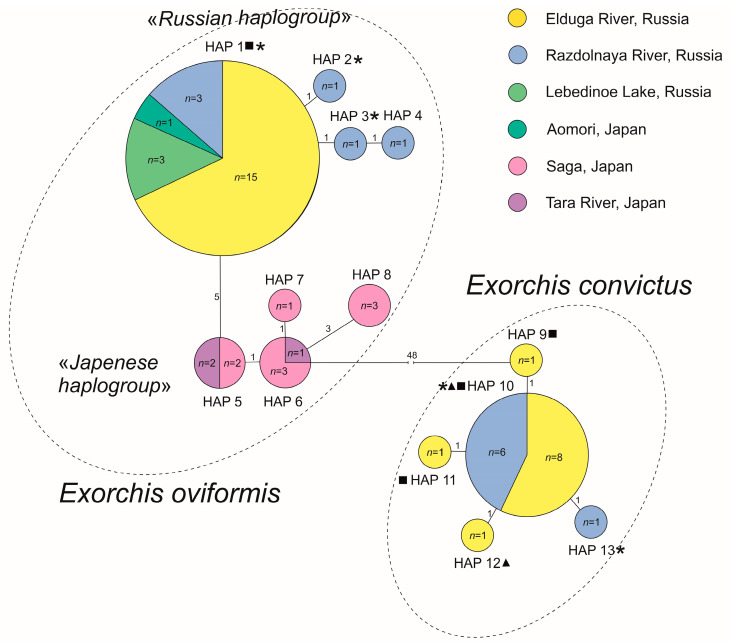
Minimum spanning tree (MST) based on the *cox1* mtDNA gene sequences for *Exorchis* spp. *Hap*—haplotype; *n*—number of sequences in the haplotypes. Square, triangle, and star are parasites from catfish 1 from the Elduga River, catfish 2 from the Elduga River, and catfish from the Razdolnaya River, respectively.

The Minimum spanning tree (MST) reconstruction differed for each species (Figure 3). It has two haplogroups for *E. oviformis*, one of which includes samples from Japan (4 haplotypes), and the other includes samples from Russia and one specimen from Japan (4 haplotypes). *Exorchis convictus* joined five haplotypes and formed a separate haplogroup. Each of the haplogroups has a star-like structure and consists of an ancestral haplotype, which is determined by their location in the center and the largest number of connections with other haplotypes. The other haplotypes differ from the ancestral ones insignificantly by 1–3 mutational steps.

## 4. Discussion

In the samples obtained for both representatives of the genus *Exorchis*, the mitochondrial marker (the *cox1* gene) revealed low genetic variability. The results also confirm the separation of these two closely related species despite sharing the same common definitive hosts and habitats, *E. oviformis* and *E. convictus*, with a genetic distance of 7.1%. These values are comparable to interspecies genetic distances within Opisthorchiidae Looss, 1899, and Heterophyidae Leiper, 1909 [12,13].

Notably, parasites from the Razdolnaya River cohabitate with the same definitive host, and both trematodes share the intestine of the same hosts, as seen in the samples from the Elduga River. Unfortunately, inadequate data from the Lebedinoe Lake (Russia), where insufficient adult individuals were collected, precluded conclusive findings. In contrast, the coexistence of both studied species in one definitive host was also not detected in Japan, considering that most of the samples in this country were obtained from intermediate hosts (fish). Given that different trematodes might spread through various intermediate hosts, it is likely that other members of the genus were not sampled.

In a similar observation using a single host by closely related parasites, Razo-Mendivil and colleagues [14] reported cohabitation in *Tabascotrema verai* Lamothe-Argumedo and Pineda-López, 1990, from the family Cryptogonimidae. The authors concluded that *T. verai* is a complex of cryptic species that use the same definitive host, *Petenia splendida* Günther, 1862. We presume that this could be generally typical for various Cryptogonimidae family representatives, since comparable cases have been reported in other studies in this family of trematodes [14,15,16]. Our findings support the complexities in trematode interactions that have yet to be fully uncovered, considering that the nuclear markers not always distinguish cryptic species. This hypothesis is supported by the low values of the genetic distances between the *T. verai* clades for nuclear markers (from 0.08% to 1.2% and from 0.3% to 0.4% for the ITS rDNA region and the 28S rRNA gene, respectively). Moreover, the average distance between clades, according to the mitochondrial *cox1* marker, revealed higher genetic distances (7.7–11.6%) between different pairs of clades, including cryptic *Tabascotrema* species [14]. Therefore, sequences of mitochondrial genes for every parasite in the family Cryptogonimidae that were extracted from naturally infected hosts are equally required for the validity of the results and to detect the occurrence of cryptic species.

Also, a population analysis performed for *T. verai* divided all haplotypes into three groups, which corresponded to clades on the phylogenetic tree according to *cox1* data, which revealed neither a geographical connection nor a correlation with watersheds [14]. But some haplotypes of different haplogroups were found in the same places, while only two haplotypes were common in individuals from different localities, and three haplotypes included specimens from the same locality. The researchers hypothesized that the high proportion of unique haplotypes found in various haplogroups is a consequence of isolation, possibly because of their intermediate or definitive hosts (or both) colonizing individual reservoirs, where some of them have undergone speciation. Additionally, the authors assumed that, since there was no connection between haplogroups and geographical location, each line of parasites could use different types of first and intermediate hosts [14].

Similar results have previously been obtained for haplogroups of *E. oviformis*, represented by samples from Japan and Russia, between which genetic differences formed because of isolation processes [1]. Our current work highlights the possibility of isolation of this cryptogonimid species in the southern part of the Russian Far East. Moreover, the low genetic variability observed in both *E. oviformis* and *E. convictus* may indicate a potential population bottleneck or limited gene flow. Consequently, geographic isolation, founder effects, or strong selection during infection might have contributed to the phenomenon of reduced levels of genetic variation. Similar patterns were seen in *T. verai* as reported by Razo-Mendivil et al., implying that these characteristics could be Crytogonimidae family traits.

The star-like haplotype structure of the *cox1* mtDNA gene (Figure 3) indicates a low diversity in both *E. oviformis* and *E. convictus*, suggesting a potential population bottleneck. This observation is consistent with the L-shaped unimodal graph of mismatch distribution for *E. convictus* [17]. Contrary to this observation, the shape of the observed mismatch distribution for *E. oviformis* is multimodal, suggesting a stable population. However, it is necessary to note the low variability within the separate haplogroups in the analysis. It is likely that the peaks were formed by each of the haplogroups within the species, which includes samples only from Russia or Japan. Compared with the data from the study of Solodovnik et al. [1], six new haplotypes have emerged. Nevertheless, a significant portion of the newly obtained samples clustered within ancestral haplotypes.

Despite the larger sample size of this study compared to that of our previous work [1], low genetic diversity was rediscovered for both species, *E. oviformis* and *E. convictus*. Previously, similar results were obtained for *Metagonimus suifunensis* Shumenko, Tatonova & Besprozvannykh, 2017, a species from the related family Heterophyidae circulating in the same area of the southern part of the Russian Far East [18]. In our earlier study, we suggested that one reason for this level of variability may be a recent decline in the population of one of its hosts [1]. To determine which host could have influenced the abundance of species of the genus *Exorchis*, it is necessary to assess the level of variability in each of the hosts that participate in their circulation. However, we believe it is also most necessary to pay attention to the first and definitive hosts, to which these trematodes have more specificity. Host variability directly influences host–parasite interactions; as defense mechanisms against helminths differ by complexity, this can lead to the extinction of parasites or their hosts due to directional selection [19]. It is also possible that abiotic factors influence the variability in the host and the regulation of its abundance, which in turn affects fluctuations in the number of parasites [20,21]. Another explanation may be that local populations of trematodes have low rates of gene flow due to the geographic isolation of their definitive hosts [22]. It is possible that in other more distant populations, parasites of both species will have a different set of haplotypes and a higher level of variability, which may provide new data on their intraspecific structure. To confirm the last assumption, we propose that, in future studies, broader genetic marker analyses and investigations of varied host populations across an expanded geographical scope are necessary to elucidate the complexities of the *Exorchis* species’ genetic variability and evolutionary dynamics.

Nevertheless, by studying parasites from a naturally infected definitive hosts, we have actually analyzed the entire set of parasites that have accumulated in intermediate hosts within the water body where the catfish lives. Observing that second intermediate hosts can be infected multiple times from their first intermediate hosts, which usually carry only clones of one parasite, and our finding of two species of parasites in the definitive host and different haplotypes within each individual species, we hypothesize that several second intermediate hosts from different places took part in the formation of the parasite population within the definitive hosts. Moreover, all catfish in this study received their parasite pool by eating different intermediate fish hosts. In any case, if the variability in the parasite population was higher, it would be possible to obtain a different set of haplotypes in each individual host that was infected naturally independently of each other. Thus, in our opinion, the sample size of host individuals has little effect on the results, and the data obtained can be a basis for further research.

It is also not excluded that the low variability obtained within both species in this study may be a consequence of competitive interactions between these parasites of the same genus. In such an instance, we assume that they will be less likely to cooperate with unrelated individuals or that there will be a conflict of interests [23]. Interspecific competition among trematodes for host resources could negatively affect the fitness of parasites. In this case, one species consumes or secretes various substances that hinder the growth and development of a competitive organism [24]. In the same vein, we believe that parasites of the genus *Exorchis* compete for access to host resources among themselves. As a result of such competition, a decrease in the population is likely to occur, which could in turn lead to a decrease in genetic diversity.

Furthermore, the observed low genetic diversity in *E. oviformis* and *E. convictus* could impact their long-term survival and adaptability, considering the limited ability to evolve in response to changing niche conditions, disease outbreaks, or host defenses, making them more susceptible to a possible extinction. Moreover, this could disrupt the ecological balance within the catfish populations they infect. In addition, if both species impact each other’s genetic variability, as has been reported by Mouritsen and Elkjær [24], it adds a complex layer to their evolutionary trajectory. Hence, the probable interspecies interactions could drive co-evolutionary adaptations, potentially leading to increased parasite specialization or, contrastingly, reduce their virulence. As a result, understanding the interplay between these parasites, their host, and the wider ecosystem by broadening the sampling density and coverage over a larger geographic area while carefully setting parasite boundaries is crucial for predicting long-term survival and their potential effects on biodiversity and ecological stability.

## 5. Conclusions

The observed low genetic variability in the *cox1* gene for both *Exorchis* species, despite their co-habitation, suggests a potential for limited gene flow or recent population bottlenecks. However, making generalized conclusions is hindered by our sample size. While *Exorchis oviformis* and *E. convictus* exhibit distinct genetic signatures, the low genetic diversity might be influenced by factors such as limited dispersal, founder effects, or strong selection during infection, leading to a limited few of *Exorchis oviformis* and *E. convictus* in the sampled locations. As a result, the interspecies interactions could potentially increase the parasite specialization or reduce their virulence. Thus, future research with expanded sampling across a wider range of host individuals, elucidation of morphological variations and geographic localities, as well as a broader selection of genetic markers, including both mitochondrial and nuclear DNA, could potentially help comprehensively explain the evidence of cryptic species, long-term survival, genetic diversity, and ecological stability patterns. Likewise, these insights could inform One Health approaches in aquaculture and natural ecosystems.

## Figures and Tables

**Table 1:** The *cox1* mtDNA sequences of *Exorchis* spp. analyzed in this study and other related information.

Species/ Developmental Stage	Locality			Length, bp	*n*	Accession Numbers	Reference
	Country	Region	Water Body				
*Exorchis oviformis*, adult	Russia	Primorsky Region	Elduga River	715	16	OR995367- OR995382	This study
			Razdolnaya River	715	2	OR995383, OR995384	This study
				753	3	MW022453- MW022455	[1]
			Lebedinoe Lake	753	3	MW022450-MW022452	[1]
*Exorchis oviformis*, metacercaria	Japan	Kyushu Island	Tara River	753	3	MW022447-MW022449	[1]
			Saga	813	9	MW014273-MW014281	[1]
		Honshu Island	Aomori	804	1	MW014285	[1]
*Exorchis convictus*, adult	Russia	Primorsky Region	Elduga River	715	11	OR995385- OR995395	This study
			Razdolnaya River	715	1	OR995396	This study
				759	6	MW022456-MW022461	[1]

*n*, number of determined sequences.

## Data Availability

The data presented in this study are available on request from the corresponding author.
